# Remission of lymphoblastic leukaemia in an intravascular fluidic environment by pliable drug carrier with a sliding target ligand

**DOI:** 10.1038/srep40739

**Published:** 2017-01-17

**Authors:** Donghyun Jang, Yeong Mi Lee, Jaehyun Lee, Junsang Doh, Won Jong Kim

**Affiliations:** 1 Center for Self-Assembly and Complexity, Institute for Basic Science (IBS), Pohang 37673, Republic of Korea; 2 Department of Chemistry, Pohang University of Science and Technology (POSTECH), Pohang 37673, Republic of Korea; 3 School of Interdisciplinary Bioscience and Bioengineering (I-Bio), Pohang University of Science and Technology (POSTECH), Pohang, 37673, Republic of Korea; 4 Department of Mechanical Engineering, Pohang University of Science and Technology (POSTECH), Pohang, 37673, Republic of Korea

## Abstract

A polyrotaxane-based nanoconstruct with pliable structure carrying a chemotherapeutic drug was developed for targeting circulating lymphoblastic leukaemia cells in a fluidic environment of blood vessels *in vivo*. By introducing lymphoblast targeting aptamer DNA through cyclodextrin, threaded in poly(ethylene glycol) as polyrotaxane, target aptamer slides along the long polymeric chain and actively search for target ligand, leading to active targeting in dynamic fluidic system which is enhanced by up to 6–fold compared with that of control carriers with non–sliding targeting ligands. Moreover, the drug carrier was made stimuli-responsive by employing i-motif DNA to selective releases of its payload at intracellular acidic condition. These combined features resulted in the effective remission of lymphoblastic leukaemia both *in vitro* and in dynamic blood vessels *in vivo*.

Lymphoblastic leukaemia is characterized by the uncontrolled production of immature lymphocytes^1^,^2^. These cancerous lymphocytes, also known as lymphoblasts, spread from the bone marrow where they originate, to various organs such as the lymph nodes, liver, spleen, or central nerve system through bloodstream^3^. Of the different lymphoblastic leukaemias, acute lymphoblastic leukaemia (ALL) progresses rapidly, and may lead to death within several months if not treated appropriately^3^,^4^. Chemotherapy, which is widely used treatment for ALL^5^,^6^,^7^, is associated with side effects that are often critical to patients. Chemotherapeutic agents tend to target highly proliferating cells^8^, including numerous healthy cells, in the cardiac tissues^9^, immune system^10^, liver^11^, etc. Therefore, intense chemotherapy may damage healthy organs, which may be fatal. This is because chemotherapeutic agents are passively infused into the body and are not specifically targeted toward cancer cells that circulate in blood vessels.

To overcome these drawbacks, studies have focused on targeted nanoparticle–based drug delivery systems with multiple functions^12^,^13^. Active targeting can be achieved by conjugating specific ligands such as small molecules^14^, proteins^15^,^16^ and aptamers^17^,^18^ to nanoparticles. This strategy selectively directs nanoparticles toward target cells and enhances their cellular uptake to maximize the therapeutic effects of the loaded drug while exerting minimal side effects on normal healthy cells. Unlike the targeting of static solid tumour tissues, targeting of rapidly circulating ALL lymphoblasts requires an intelligent drug carrier that can actively search and bind to these cells with high binding affinity.

To fulfil the requirement, we used polyrotaxane (PR), which has a unique structure composed of mechanically interlocked ring–shaped macrocycles such as cyclodextrin (CD) threaded into a long polymeric backbone^19^, as a base component for developing a pliable drug carrier. CD was modified with an aptamer DNA that has a strong binding affinity toward proteins on target cell surface. Aptamer DNA–CD component placed along the polymeric polyethylene glycol (PEG) backbone of PR has high degree of sliding freedom^20^,^21^,^22^,^23^,^24^. This unique sliding ability increases the probability of the aptamer DNA to bind to target molecules on cancer cells and allows attached ligands to migrate toward other target molecules to form multimeric bonds, leading to the formation of a robust binding between the carrier and target cells^21^,^22^,^25^.

Along with employing sliding ligand for enhanced targeting ability to target cells, stimuli–responsive behaviour that releases drug molecules only in the desired region decreases the toxicity of chemotherapeutic agents in non-target region^26^. Different intracellular conditions such as pH^14^,^27^, reductive potential^16^, enzymes^28^, and small molecules^29^ have been intensively examined as trigger discharging chemotherapeutic agents in programmed environments. We used pH–responsive “i–motif” DNA^30^ to induce the stimuli–responsive behaviour of the drug carrier^31^,^32^. Unique double–stranded DNA (dsDNA) structure formed by the i–motif DNA and its complementary DNA responds to intracellular acidic environment^33^ and releases intercalated anti–cancer agent doxorubicin (DOX) by inducing its dissociation from the dsDNA^27^. By integrating these functions, we have developed an effective lymphocyte targeting drug carrier in circulating bloodstream environment (Fig. 1).

## Results

### Synthesis and characterization of pliable drug carrier with a sliding target ligand

**Figure 2:**
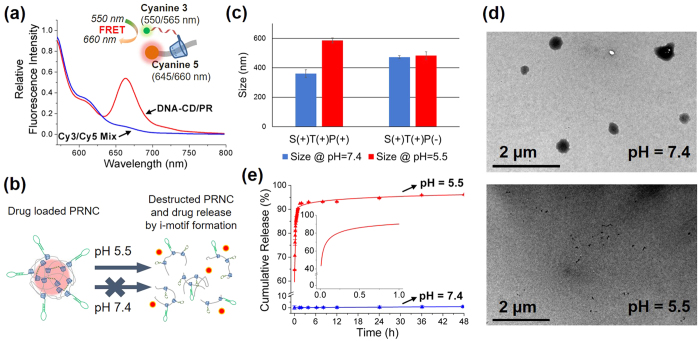
Characterization of PRNC. (**a**) Formation of the PR structure was confirmed by the increase in the FRET signal of Cy3 and Cy5. Red line indicates fluorescence signal from polyrotaxane structure of Cy3-DNA-CD and Cy5-PEG, while blue line shows that of their simple mixture. (**b**) Schematic illustration of destructed PRNC upon acid stimuli and drug release. (**c**) Size of PRNC and non i-motif control monitored by DLS at different pH. (**d**) Destruction of PRNC upon acid stimuli is shown by TEM image in different pH condition. (**e**) Cumulative release of DOX from the PRNC was monitored for 48 h in a buffer of pH = 5.5 (red curve) and pH = 7.4 (blue curve). Error bar indicated by triplicate experiment.

The PR–based stimuli–responsive flexible drug carrier was developed by modifying CD, supramolecule with cyclic structure, and by threading it selectively into PEG. Commercially available αCD was thiolated to αCD–SH by using a method described previously (Supplementary Fig. 2)^34^,^35^. Next, αCD–SH was conjugated with amine modified i–motif DNA or aptamer DNA (Supplementary Table 1) by using a heterobifunctional crosslinker (Supplementary Fig. 1a,3). PR and fluorescent PR were prepared using a capping procedure reported previously (Supplementary Fig. 1b,c)^19^. After DNA–CD was threaded into PEG, successful formation of the PR structure was confirmed by measuring the increase of fluorescence resonance energy transfer (FRET) signal between cyanine 3 dye (Cy3, ex = 550 nm; em = 565 nm) at the 3′ end of i–motif DNA and cyanine 5 (Cy5, ex = 645 nm; em = 660 nm) at the end of PEG (Fig. 2a). Formation of polyrotaxane structure restricts the distance between the Cy3-DNA and Cy5-PEG by mechanically interlocked structure. The inclusion of the Cy3-DNA-CD into PEG enables both dyes to be sufficiently close to induce FRET. In contrast, the simple mixture of Cy3-DNA and Cy5-PEG cannot induce close approach of two fluorescence dyes. Therefore, the increased FRET signal in 660 nm indicates the successful formation of polyrotaxane structure. Moreover, the successful formation of PR was also confirmed by the molecular weight increase of CD-DNA/PR by gel permeation chromatography (GPC) (Supplementary Fig. 4).

PRs each containing i-motif DNA or its complementary DNA were prepared by following the method discussed above. PRs were annealed to form a polyrotaxane-based nanoconstruct (PRNC) by hybridizing i–motif DNA–CD with complementary DNA–CD. This double-stranded DNA formation acts as a crosslinker to form the structure of the PRNC. The obtained PRNCs were approximately 400 nm in size as indicated by dynamic light scattering (DLS) size measurement data (Supplementary Fig. 5). Transmission Electron Microscopy (TEM) image of PRNCs was well correlated to DLS data (Supplementary Fig. 7). The NCs were stable in 10% serum condition for 24 h (Supplementary Fig. 6), meaning its suitability for biological applications. The double stranded DNA (dsDNA) of the i–motif DNA and complementary DNA dissociates in acidic environment because of the formation of characteristic quadruplex structure by the i–motif DNA in this environment, and it was confirmed by circular dichroism spectra (Supplementary Fig. 8).

To verify the pH–responsive destruction of the PRNCs (Fig. 2b), the size of the PRNCs was measured by performing DLS also in an acidic buffer (Fig. 2c). The size of the PRNCs increased under the acidic condition, indicating that dissociation of the dsDNA because of the formation of the i–motif structure in the acidic buffer loosened the PRNC structure, thus swelling it slightly. pH-responsive dissociation of PRNC was also confirmed by TEM image in different pH condition (Fig. 2d).

Change in the fluorescence of DOX was monitored to study the drug loading and releasing behaviour of the PRNCs. Intercalation of DOX into the base pairs of dsDNA quenches its characteristic fluorescence (Supplementary Fig. 9). The drug loaded PRNCs underwent haemolysis assay to assure the safety of PRNC when treated into biological systems. There was no significant damage occurred to the red blood cells observed (Supplementary Fig. 10). Release of loaded drug molecules from the PRNC was monitored at different pH levels. Control experiment with a neutral buffer (pH = 7.4) showed only 1% cumulative drug release during 48 h (Fig. 2e, blue curve). However, when the pH was decreased to 5.5, a burst release of DOX was observed. Incubation at pH 5.5 for 1 h released 90% DOX from dsDNA (Fig. 2e, inset), while incubation at the same pH for 48 h released up to 95% DOX (Fig. 2e, red curve). In contrast, PRNC lacking the i–motif DNA sequence did not significantly release DOX upon acidic stimuli (Supplementary Fig. 11). These results confirm that acidic intracellular environment triggers the release of intercalated drug from its carrier within a short time.

### Evaluation of the targeting ability of PRNC with sliding ligand in cultured cells

While the i–motif DNA provides structural rigidity and stimuli–responsive drug delivery by the PRNC, the aptamer DNA allows PRNC to target specific cells. In present study, we used protein tyrosine kinase 7 (PTK7) aptamer^36^,^37^ because PTK7 is highly expressed on the membrane of human T lymphoblast cell line CCRF–CEM, a well–studied model of ALL. PTK7 aptamer is known to induce internalization of the nanoparticles into the cells via endocytosis^38^. By endocytosis, PRNC is internalized by the acidic vesicles called endosome which matures into lysosome, which are known to be acidified by ATP-dependent proton pump^39^,^40^. This process results in gradual decrease of vesicle’s pH up to 4.5–5.0 in lysosomal condition, which can induce the dissociation of i-motif structure of PRNC. For comparison, PTK7–underexpressing Ramos cell line was used as a control.

We hypothesized that the threaded aptamer DNA–CD will slide along the polymeric PEG chain with a high degree of freedom, thus increasing its probability of binding to target molecules on the surface of circulating cancer cells and maintaining a stronger binding affinity even in the dynamic environment of the bloodstream. To confirm this, we first investigated the uptake efficiency of the PRNC by static cells cultured *in vitro*. We prepared a series of fluorescent Cy5–labelled PRNCs with or without a targeting moiety (T+/−), sliding ability (S+/−), and pH–responsive i–motif DNA sequence (P+/−) (Supplementary Table 2). PRNC without sliding ability was prepared by introducing excess amount of bare αCDs during PR formation to fill the vacancy in PEG backbone, as previously reported that the increase of number of CDs in linear axle restricts the sliding ability of individual CDs^41^,^42^,^43^,^44^,^45^,^46^. The increase of molecular weight confirmed by GPC (Supplementary Fig. 4, red curve) showed successful incorporation of bare CDs in PEG backbone.

**Figure 3:**
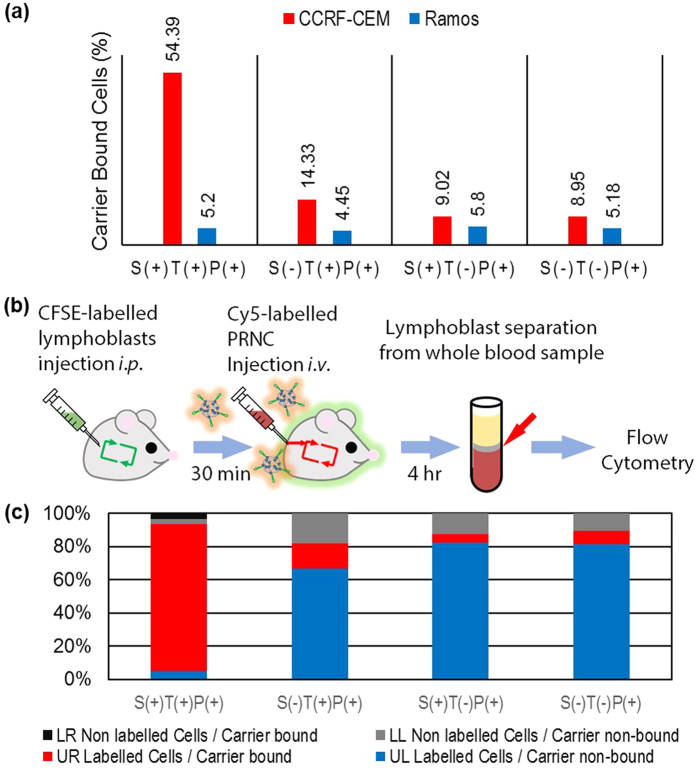
Evaluation of the targeting ability of PRNCs both *in vitro* and *in vivo*. (**a**) The percentage of carrier-bound CCRF-CEM and Ramos cells was measured by flow cytometry after treating cells with various PRNCs *in vitro*. (**b**) Illustration of the procedures of *in vivo* assessment of the targeting ability of the PRNCs. Whole blood samples of PRNC-treated mice were analysed by performing flow cytometry after injecting CFSE-labelled CCRF-CEM cells, followed by the injection of PRNCs. (**c**) Distribution of labelled CCRF-CEM cells in each quadrant is shown for mice treated with different PRNCs.

Results of *in vitro* flow cytometry (Fig. 3a, red bar) performed within 4 h after PRNC treatment showed that only S(+)T(+)P(+) PRNC effectively targeted CCRF–CEM cells. Non–sliding S(−)T(+)P(+) PRNC showed slightly enhanced cellular uptake; however, this uptake was far low compared with that of S(+)T(+)P(+) PRNC. Other PRNCs lacking the targeting moiety, i.e., S(+)T(−)P(+) or S(−)T(−)P(+) PRNC revealed similar targeting ability as non–treated negative control PRNCs. These results support our hypothesis that sliding target ligands on drug carriers show effective active targeting, which enhances their cellular uptake. However, in control Ramos cells, the various Cy5–PRNCs did not exert significantly different effects (Fig. 3a, blue bar) irrespective of their sliding properties. This may be because of the underexpression of target molecules on the surface of Ramos cells.

### *In vivo* study of the targeting ability of PRNC in dynamic blood stream

To validate active targeting of PRNCs with the sliding ligand, we conducted a series of experiments *in vivo* in dynamic mouse bloodstream environment (Fig. 3b). To minimize graft versus host rejection and confusion during analysis, we used immunodeficient Balb/c nude mice in these experiments. We designed a harsh condition by co–introducing both target circulating lymphoblasts and drug carriers into the mouse circulatory system. Target lymphoblasts were labelled with carboxyfluorescein succinimidyl ester (CFSE), a green fluorescent cell–staining dye, to differentiate them from intrinsic mouse mononuclear cells. The stained lymphoblasts were injected intraperitoneally (i.p.), followed by the injection of Cy5–PRNCs intravenously (i.v.) after 30 min. Within 4 h after the injection, whole blood was obtained from the treated mice and was analysed by performing flow cytometry. By counting the cells located in each quadrant of the dot plot, it was observed that 88% labelled lymphoblasts were successfully targeted by Cy5–PRNCs in S(+)T(+)P(+) PRNC-treated mouse (Fig. 3c). However, surprisingly low percentage of labelled lymphoblasts (range, 5%~15%) was targeted by Cy5-PRNC in S(−)T(+)P(+), S(+)T(−)P(+) and S(−)T(−)P(+) PRNC treated mice. Ramos cell-treated mice showed no significant increase in Cy5-PRNC fluorescence signal in retrieved cells (Supplementary Fig. 12). These data strongly indicate that our sliding active targeting system significantly enhances cellular uptake efficiency of drug carrier even in *in vivo* fluidic environment.

### Anti-cancer effect of PRNC against cultured lymphoblasts

**Figure 4:**
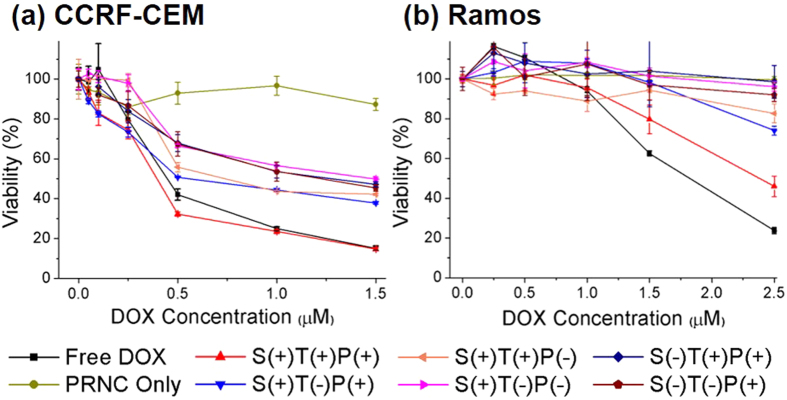
*In vitro* cell viability profile of (**a**) CCRF–CEM and (**b**) Ramos cells treated with various PRNCs.

Because enhancement of targeting ability eventually increases the therapeutic effect of a drug carrier, we examined drug–induced cytotoxicity in cultured cell lines. In addition to active targeting induced by the sliding aptamer DNA, stimuli–responsive drug release induced by the i–motif dsDNA is crucial for treating circulating leukemic cells. We treated CCRF–CEM and Ramos cells with various PRNCs and monitored the cytotoxic effect of the PRNCs by performing a standard MTT assay. S(+)T(+)P(+) PRNC exerted higher cytotoxic effects on CCRF-CEM cells (Fig. 4a) than free DOX or other control carriers (See Supplementary Table 3 for IC 50 value). PRNC lacking aptamer or i–motif DNA was less effective because it lacked the targeting ability or stimuli–responsive behaviour, respectively. As expected, PRNC lacking both aptamer and i–motif DNA had low cytotoxicity. In contrast, free DOX exerted higher cytotoxic effect on Ramos cells (Fig. 4b) than other PRNCs. This may be because PRNCs were not effectively transported into cancer cells with assistance of targeting aptamers. Aptamer–modified S(+)T(+)P(+) PRNC exerted the highest cytotoxic effects on Ramos cells because of low but positive expression of PTK7 on these cells. However, other PRNCs showed no significant cytotoxicity even at the highest concentration of DOX.

### Remission of lymphoblastic leukaemia under intravascular fluidic environment in mouse model

**Figure 5:**
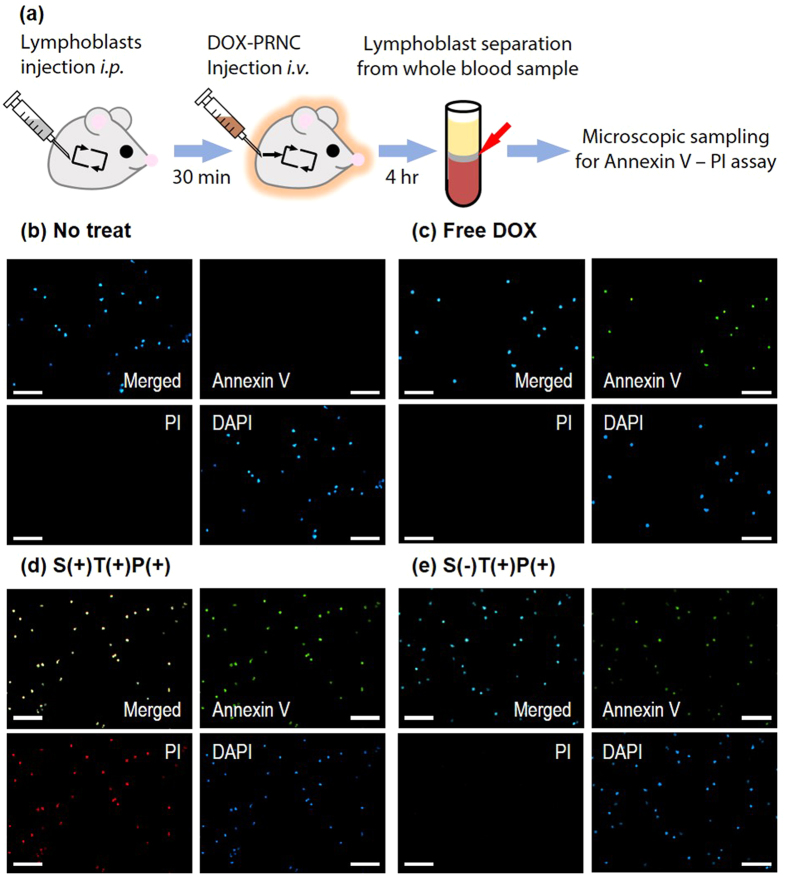
Evaluation of the cytotoxic ability of PRNCs *in vivo* by Annexin V–PI apoptosis assay of lymphoblasts. (**a**) Illustration of the overall experimental procedure. Fluorescent microscopic image of lymphoblasts (**b**) not treated, or treated with (**c**) free DOX, (**d**) S(+)T(+)P(+) PRNC, and (**e**) S(−)T(+)P(+) PRNC. (Scale bar = 100 μm).

Favourable *in vitro* results obtained using the pH–responsive flexible drug carrier prompted us to apply it *in vivo* in a mouse model. To design an *in vivo* model for studying the remission of leukaemia in a dynamic bloodstream environment, we introduced CCRF–CEM lymphoblasts i.p. into a mouse blood vessel, followed by the injection of DOX–loaded PRNCs i.v. after 30 min. At 4 h after the PRNC injection, whole blood was obtained from the treated mice and was analysed by Annexin V/propidium iodide (PI) assay. (Figure 5a) We observed that S(+)T(+)P(+) PRNC–treated mice had higher percentage of apoptotic/necrotic CCRF-CEM cells as indicated by a bright illumination of Annexin V signal in a fluorescent image, than control mice (Fig. 5b,c,d,e; Supplementary Fig. 13). The fluorescence of the region of interest was quantified using an image processing software (Supplementary Table 4). These results confirm that the drug delivery system with the sliding targeting moiety and stimuli–responsive behaviour effectively killed target lymphoblasts in a fluidic environment *in vivo*.

## Discussion

By adopting the PR as structural basis of the nanoparticle system, we have succeeded in the introduction of linear mobility to the targeting moieties. Our experimental results demonstrate that pliability of targeted drug delivery carrier has increased the lymphoblast remission both *in vitro* and *in vivo* model, as we expected. Especially, in the *in vivo* model we adopted to verify the anticancer activity of our drug carrier has simulated complicated obstacles such as fluid velocity, non-specific interactions with other cells and proteins, acute toxicity in organ functions, and rapid clearance of nanoparticles. These may have disturbed the carrier system to malfunction in practice, but our PRNC has overcome these barriers, showing the chance to successful application in more complicated systems.

In summary, we developed a pliable drug delivery carrier by using a polyrotaxane structure that allowed the sliding of the targeting ligand, thus enhancing the active targeting efficiency even in an *in vivo* fluidic bloodstream environment. In addition, we used an acid stimuli-responsive system for the programmed delivery of a drug molecule. As expected, the sliding flexibility of the aptamer DNA greatly increased the adhesion of the PRNC to target lymphoblasts. These results suggest that this drug carrier with the sliding target ligand can be used for actively targeting circulating cells such as circulating tumour cells in a dynamic bloodstream environment.

## Methods

### Materials and reagents

Amicon® Ultra-0.5 centrifugal filter device was purchased from Merck Millipore (Darmstadt, Germany). Cyanine5 NHS ester was purchased form Lumiprobe Corp. (Hallandale Beach, FL). Ficoll-Paque PLUS density gradient was purchased from GE Healthcare (Piscataway, NJ). All other chemicals were purchased from Sigma-Aldrich (St. Louis, MO). Purchased commercial reagents were used without further purification. All DNAs were purchased from IDT Inc. (Coralville, IA). The details of DNA sequences used in the experiments are shown in Table S1.

### Instrumentations

The ^1^H-NMR spectrum was obtained using 500 MHz NMR spectrometer (DRX 500MHZ, Bruker, Germany) and processed using MestReNova (Version 7.1.2, Mestrelab Research S.L., Santiago de Compostella, Spain) and mass spectrum was recorded by LTQ Velos dual ion trap mass spectrometer (Thermo Scientific, Bremen, Germany). High performance liquid chromatograph (HPLC) and Gel permeation chromatography (GPC) data was obtained by HPLC/GPC system (UFLC Prominence, Shimadzu Corp., Kyoto, Japan) and processed with LC Solutions software (Version 1.22 SP1, Shimadzu). Hydrodynamic volumes and surface charges of materials were obtained by a Zetasizer Nano (Malvern Instruments, Malvern, UK). TEM image was obtained by JEM-1011 (JEOL Inc., Peabody, MA). The fluorescence spectra were measured using a fluorescence spectrometer (LS55, Perkin Elmer, Waltham, MA). Flow cytometry analysis data was obtained by FACSCalibur (Becton Dickinson, San Jose, CA) and processed with Cell Quest software (Becton Dickinson).

### Synthesis of Mono-6-mercapto-6-deoxy-α-cyclodextrin (αCD-SH)

αCD-SH was prepared starting from the Mono-6-(p-toluenesulfonyl)-6-deoxy-α-cyclodextrin (αCD-OTs). αCD-OTs (100 mg, 89 μmol) and thiourea (200 mg, 2.63 mmol) were dissolved to 80% aqueous methanol then refluxed at 110 °C with stirring for 24 h. 10% aqueous sodium hydroxide was then added to the reaction vessel after solvent evaporation with rotary evaporator, and the reaction mixture was stirred for 24 h at 40 °C. The pH of reaction mixture was adjusted to 2 by adding 1 M hydrochloric acid (aq), 2 mL trichloroethylene was added and stirred for another 10 h in RT. Reaction mixture was dried under reduced pressure after removal of trichloroethylene layer. The remaining yellow solid was redispersed in 5 mL N,N-dimethylformamide to filter off the precipitate. Solvent of the filtrate was evaporated until the mixture became highly viscous, then precipitated by adding excess amount of cold acetone. Precipitate was filtered and washed several times with cold acetone and dried under high vacuum with mild heat to remove the residual solvents. Product was analysed by ^1^H NMR (Figure S2) and ESI-MS spectroscopy (Calculated for C_36_H_59_O_29_S^−^Na^+^: 1010.28, Found 1010.72). The yield was calculated by ^1^H NMR. (40.9 mg, 46%).

### Chemical conjugation of CD to DNAs

DNAs were diluted into 1 mM solution prior to further experiments by adding appropriate amount of buffer solution. Amine-modified DNAs were crosslinked with SPDP (Thermo Scientific, Waltham, MA) in prior to the attachment with αCD-SH. In carbonate-Na buffer (0.5 M, pH = 9.2), DNA-NH_2_ was mixed with excess amount of SPDP, then reacted overnight with constant vortexing. Reaction mixture was purified and concentrated by centrifugal filter with 3 K MWCO. The concentrated product was redispersed in appropriate amount of buffer, then added excess amount of αCD-SH. The reaction mixture was again reacted for overnight with constant vortexing, and was purified and concentrated by centrifugal filter with 3 K MWCO. Final reaction mixture was lyophilized for further usages. Synthesized DNA-CD was confirmed by HPLC analysis (Figure S3).

### Formation of polyrotaxane nanoconstruct (PRNC)

To synthesize two different types of polyrotaxanes, we first synthesized poly-pseudorotaxanes. Series of polyrotaxanes which are required for construction of various PRNCs possessed 9 equivalent of binding sequence and 1 equivalent of targeting sequence in each diamino-poly(ethyleneglycol) (20 KDa, Nanocs Inc. New York, NY). The components were mixed in phosphate buffered saline (PBS, Bioneer, Korea) and incubated overnight at 4 °C after sonication for 1 h. For non-sliding control groups, we added excess αCD to this mixture, then incubated overnight after sonicating another 1 h. Capping procedure followed by adding excess amount of 1-fluoro-2,4-dinitrobenzene then vortexing overnight in RT. Reaction mixtures were lyophilized and redispersed in PBS. Synthesized polyrotaxanes were annealed to form PRNCs by incubation for 10 min at 90 °C then overnight at 4 °C. Annealed PRNCs were again lyophilized and redispersed in PBS to make desired concentration for further experiments.

### Serum stability test of PRNC

Stability of PRNC under serum condition was tested by following procedures. PRNC was incubated in 10% fetal bovine serum (FBS, Hyclone) containing Roswell Park Memorial Medium (RPMI-1640, Hyclone) at 37 °C up to 24 h. The hydrodynamic size of PRNC was measured by DLS without serum or with serum at 1 h, 2 h, 6 h and 24 h.

### Haemolysis test of PRNC

Release of haemoglobin from lysed erythrocytes were evaluated by measuring the UV absorbance in 540 nm wavelength of each samples. Fresh mouse blood was diluted 10 times in PBS for haemolysis test. To 380 μL diluted mouse blood, 20 μL of each sample either containing Triton X-100, PBS, NC Only or 80 μM DOX (Free DOX, S(+)T(−)P(+) PRNC/DOX, S(+)T(+)P(−) PRNC/DOX) were added. These mixtures were incubated at 37 °C for 6 h. Incubated mixtures were centrifuged at 2000 rpm for 15 min, then supernatants were collected to measure the absorbance. Triton X-100 was utilized as positive control (100% haemolysis).

### Drug loading and releasing test

Before the drug loading and releasing tests, fluorescence of DOX (ex = 495 nm; ex = 595 nm) was measured at different pH conditions (7.4 and 5.5). Intercalation of DOX in i-motif DNA was observed by measuring the intensity of DOX fluorescence after mixing with appropriate concentrations of PRNCs. Release of DOX from PRNCs was measured by obtaining fluorescence of single point (λ = 595 nm) continuously during 48 h for every 1 min.

### Cell culture and animal model

CCRF-CEM is an acute T-lymphoblastic leukaemia cell line derived from human peripheral blood, which overexpresses protein tyrosine kinase 7 (PTK-7) on the cell surface. Ramos is a Burkitt’s lymphoma cell line which underexpresses PTK-7. Both cell lines were obtained from Korean Cell Line Bank (KCLB) and were cultured in Roswell Park Memorial Medium (RPMI-1640, Hyclone) containing 10% fetal bovine serum (FBS, Hyclone), 100 U mL-1 penicillin (Hyclone) and 100 μg mL-1 streptomycin. Cells were cultured in 5% CO2 humidified incubator set at 37 °C. Female BALB/c Nude mice were received from POSTECH Biotech Center. All animal experiments in this study were approved by the POSTECH Biotech Center Ethics Committee and all methods were performed in accordance with the relevant guidelines and regulations.

### *In vitro* cellular uptake efficiency assay

To test the *in vitro* cellular uptake efficiency of PRNCs, CCRF-CEM or Ramos cells were counted and transferred to 24-well plates at a density of 2 × 10^5^ cells per well in 1 mL serum-containing media, then Cy5-PRNCs were treated into each well. After 4 h, the treated lymphoblasts were retrieved from the well plates and washed several times with cold PBS, then fixed with 10% neutral buffered formalin. The fluorescence level of Cy5 in each cell was measured by flow cytometry.

### *In vivo* uptake efficiency study

Prior to the *in vivo* uptake efficiency test, CCRF-CEM and Ramos cells were labelled green using the CellTraceTM CFSE cell proliferation kit (Molecular probes, Eugene, OR) according to the protocol of the manufacturer. Labelled CCRF-CEM or Ramos cells (2 × 10^6^ cells) were injected to mice i.p., then Cy5-PRNCs were injected i.v. subsequently. After 4 h of circulation, whole blood of sample-treated mice was gathered directly from the heart of the mice. Mice were immediately sacrificed after obtaining blood sample. Lymphoblasts were separated from mice whole blood by following the standard protocol of Ficoll-Paque PLUS density gradient. Lymphoblasts were washed several times with cold PBS, then fixed with 10% neutral buffered formalin. The levels of green fluorescence of cells and red fluorescence of Cy5-PRNCs were analysed by flow cytometry.

### *In vitro* cytotoxicity test

*In vitro* cell viability of PRNCs and controls was measured using 3-(4,5-dimethylthizol-2-yl)-2,5-diphenyltetrazolium bromide (MTT). CCRF-CEM or Ramos cells were counted and transferred to 96-well plates at a density of 8 × 10^3^ cells per well in 80 μL serum containing media. Treatment of each samples were performed by adding 10 μL of PBS dispersed samples to the wells. After incubating 18 h, 10 μL of MTT solution (5 mg mL-1) was added in each wells. Cells were then lysed by adding 100 μL 10% SDS aqueous solution (pH = 5.5, Bioneer, Korea) then incubating overnight. The absorbance of formazan salt at 570 nm was measured using a microplate reader (VICTOR 3-V Multilabel counter, PerkinElmer, Wellesley, MA).

### *In vivo* remission of lymphoblasts study in mouse model

For the *in vivo* cytotoxicity assay, CCRF-CEM cells (2 × 10^5^ cells) were injected to mice i.p., then samples were injected i.v. subsequently. Similar to the methods described in the *in vivo* uptake efficiency test section, lymphoblasts were separated from mice whole blood. Lymphoblasts were thoroughly washed with cold PBS, then treated with Annexin V-FITC Apoptosis Detection Kit (Sigma-Aldrich). Treated samples were made into microscopic sample by mounting on microscopic slide glass with mounting medium with DAPI (Vector Laboratories, Burlingame, CA). Images were obtained from the prepared samples with fluorescent microscope system (Eclipse Ti-E, Nikon, Kobe, Japan) equipped with CCD camera (Coolsnap MYO, Photometrics, Tucson, AZ) then processed with NIS-Elements Advanced Research software (Version 4.2, Nikon).

## Additional Information

**How to cite this article**: Jang, D. *et al*. Remission of lymphoblastic leukaemia in an intravascular fluidic environment by pliable drug carrier with a sliding target ligand. *Sci. Rep.* **7**, 40739; doi: 10.1038/srep40739 (2017).

**Publisher's note:** Springer Nature remains neutral with regard to jurisdictional claims in published maps and institutional affiliations.

## Supplementary Material

Supplementary Information

## Figures and Tables

**Figure 1:**
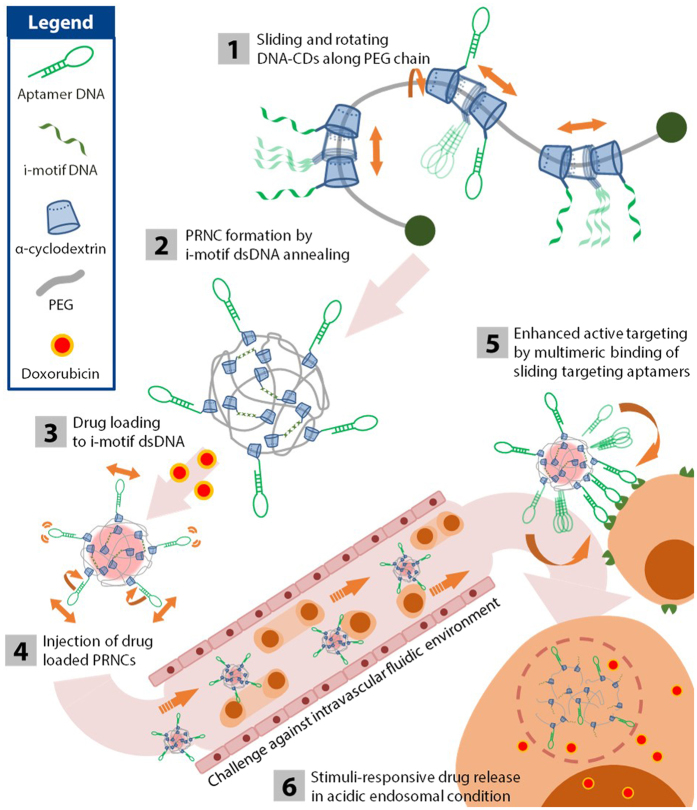
Schematic representation of the stimuli-responsive pliable drug carrier with the sliding target ligand, and the strategy for targeting lymphoblasts and drug release.
