# Supplementary material for: Remission of lymphoblastic leukaemia in an intravascular fluidic environment by pliable drug carrier with a sliding target ligand
Source: Sci Rep. 2017 Jan 17;7:40739. doi: 10.1038/srep40739 (PMC5240144; doi:10.1038/srep40739)
Supplement: Supplementary Information [file srep40739-s1.pdf]

Supporting Information

Remission of lymphoblastic leukaemia in an intravascular fluidic environment by pliable drug carrier with a sliding target ligand

Donghyun Jang[1,2], Yeong Mi Lee[1,2], Jaehyun Lee[3], Junsang Doh[3,4] and Won Jong Kim[1,2,*]

[1] Center for Self-Assembly and Complexity, Institute for Basic Science (IBS), Pohang 37673, Republic of Korea

[2] Department of Chemistry, Pohang University of Science and Technology (POSTECH), Pohang 37673, Republic of Korea

[3] School of Interdisciplinary Bioscience and Bioengineering (I-Bio), Pohang University of Science and Technology (POSTECH), Pohang, 37673, Republic of Korea

[4] Department of Mechanical Engineering, Pohang University of Science and Technology (POSTECH), Pohang, 37673, Republic of Korea

Contents

1. **Supplementary Figures ………………………………………….....S3**

Supplementary Figure 1

Synthetic schemes of DNA-CD and PRs…..…………………….……...S3

Supplementary Figure 2

Confirmation of αCD-SH by ^{1}H NMR spectroscopy………………..…..S4

Supplementary Figure 3

HPLC data of DNA-CD……………………………………….....…..S4

Supplementary Figure 4

GPC data of PEG and PRs…………………………………….……S5

Supplementary Figure 5

DLS size measurement of various PRNCs………………………………S6

Supplementary Figure 6

Serum stability of PRNC...…………S6

Supplementary Figure 7

TEM image of various PRNCs……………………………...…..……..…S7

Supplementary Figure 8

CD spectra of i-motif DNA in different pH……………………..……..…S8

Supplementary Figure 9

Drug loading properties of PRNC…………………………………..………S8

Supplementary Figure 10

Haemolysis test of PRNC and control groups…………………...…………S9

Supplementary Figure 11

Release of DOX from S(+)T(+)P(-) PRNC in acidic condition …………...S9

Supplementary Figure 12

Detailed flow cytometry data of in vivo targeting efficiency test……..…S10

Supplementary Figure 13

Additional images of *in vivo* cytotoxicity evaluation ……….……..….…S11

2. Supplementary Tables ……………….………………………....……......S12

Supplementary Table 1

Sequences of DNAs used in this experiment…………………………….S12

Supplementary Table 2

Types of PRNCs used in this experiment………………………………..S12

Supplementary Table 3

IC50 values of DOX and DOX loaded PRNCs…………....…................…S13

Supplementary Table 4

ROI fluorescence intensity calculation of microscopic images………….S13

1. Supplementary Figures

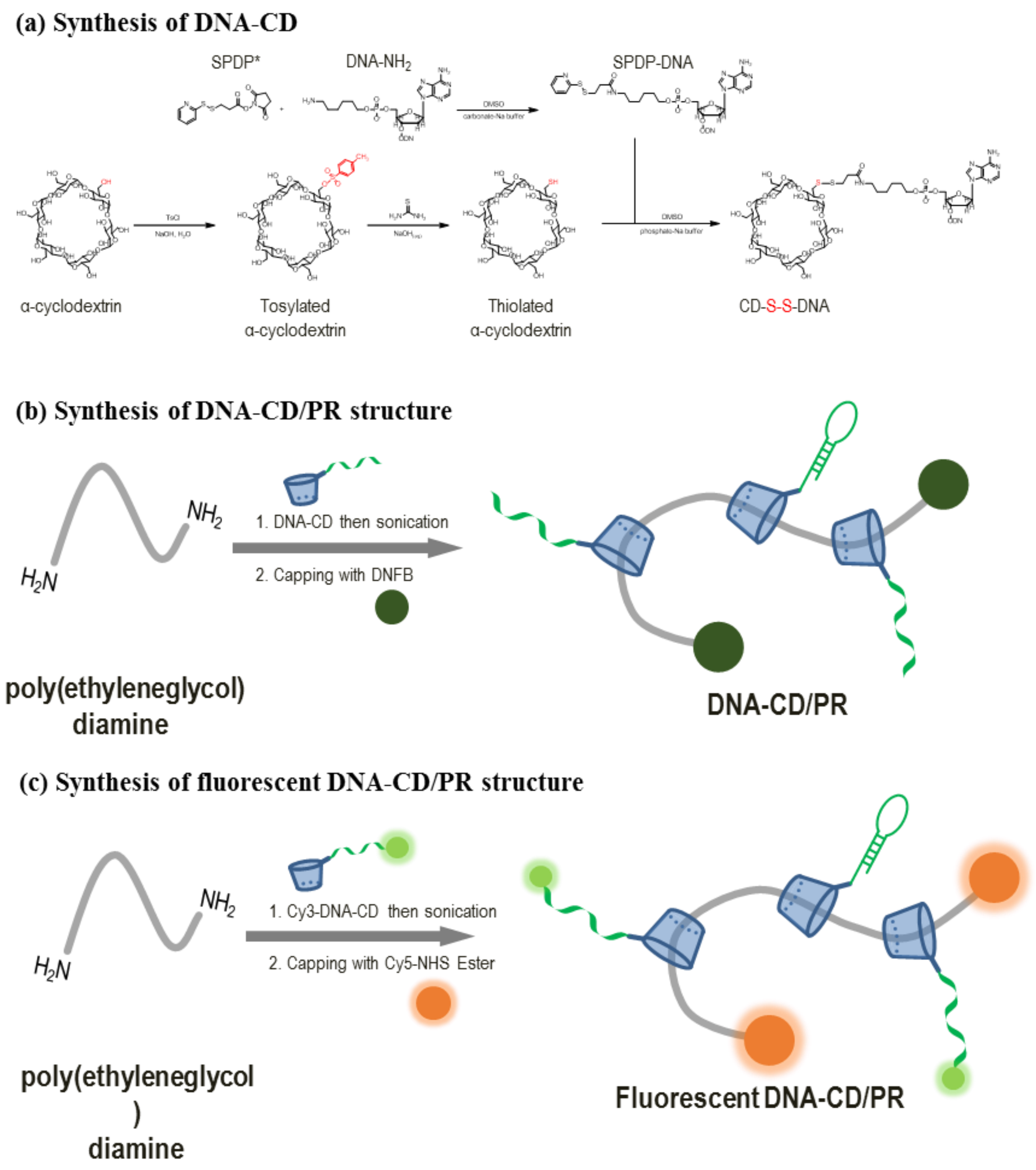

Supplementary Figure 1 (a) Experimental schemes for synthesis of αCD-SH and DNA-CD. (b) Preparation of DNA-CD/PR by capping strategy. (c) Preparation of the fluorescently labelled DNA-CD/PR.

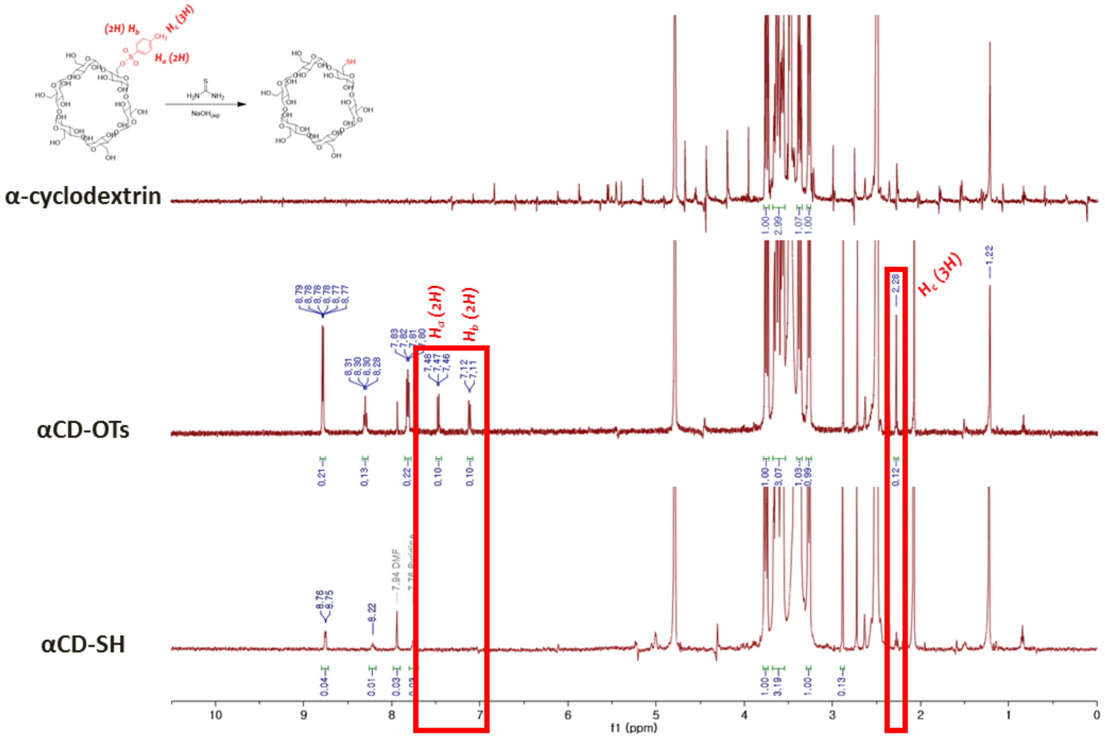

Supplementary Figure 2 Confirmation of Mono-6-mercapto-6-deoxy-α-cyclodextrin by ^{1}H NMR spectroscopy. Successful synthesis of αCD-SH was confirmed by the disappeared tosylate peaks in final NMR spectra.

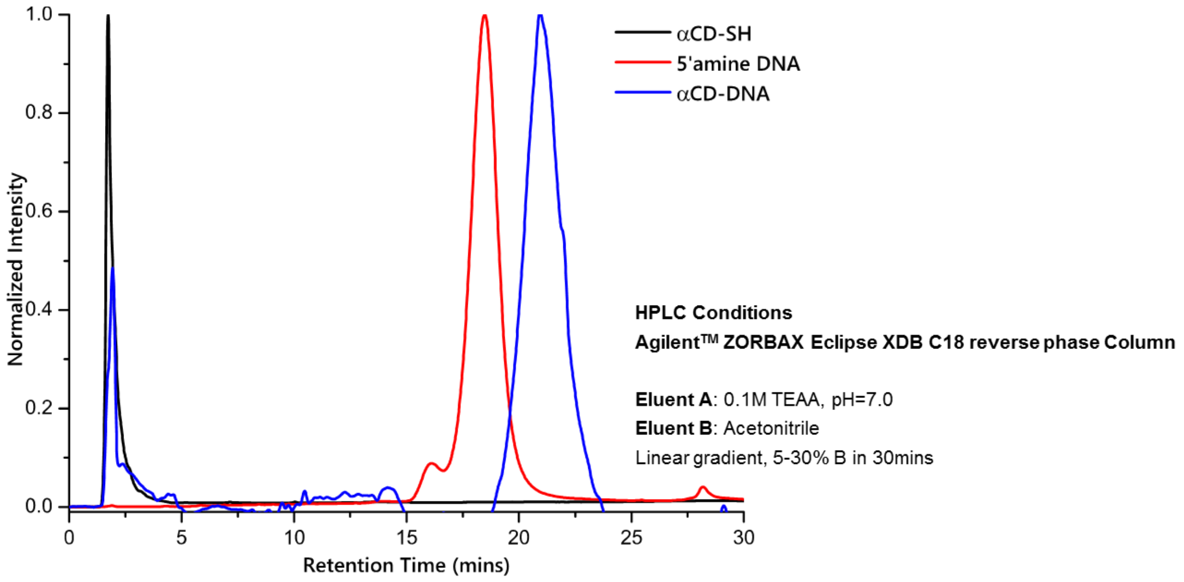

Supplementary Figure 3 HPLC data of DNA-CD.

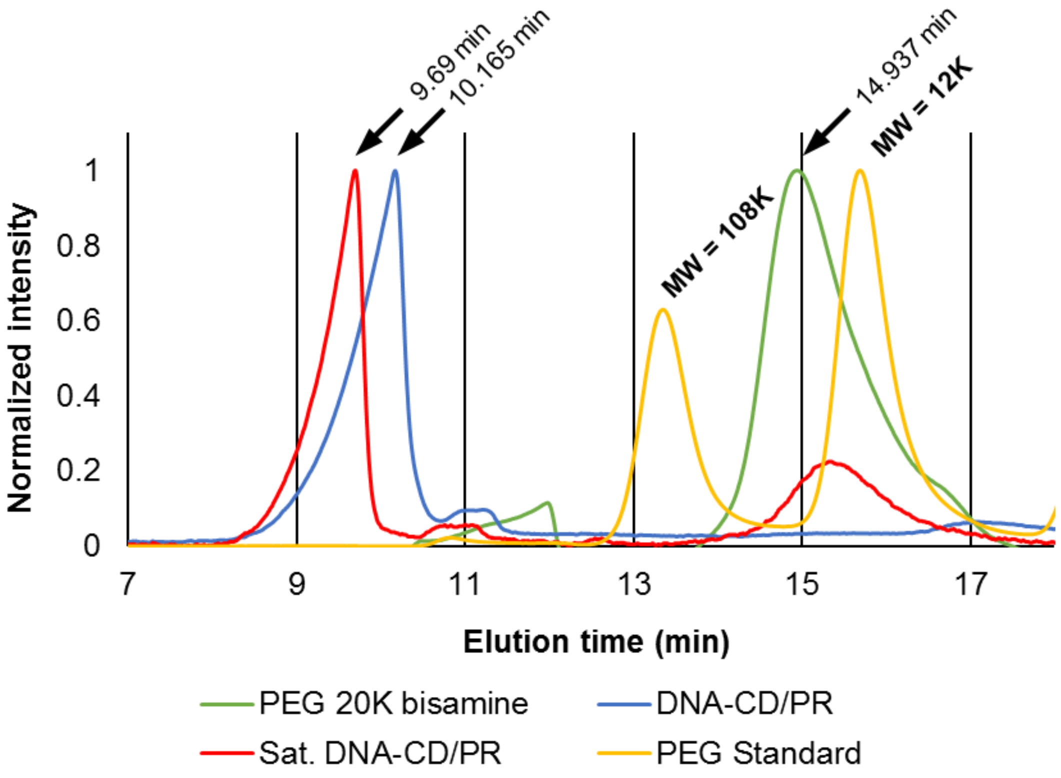

Supplementary Figure 4 GPC data of 20K PEG-$(AM)_2$, DNA-CD/PR and non-sliding Sat. DNA-CD/PR shown with calibration curve by PEG standards.

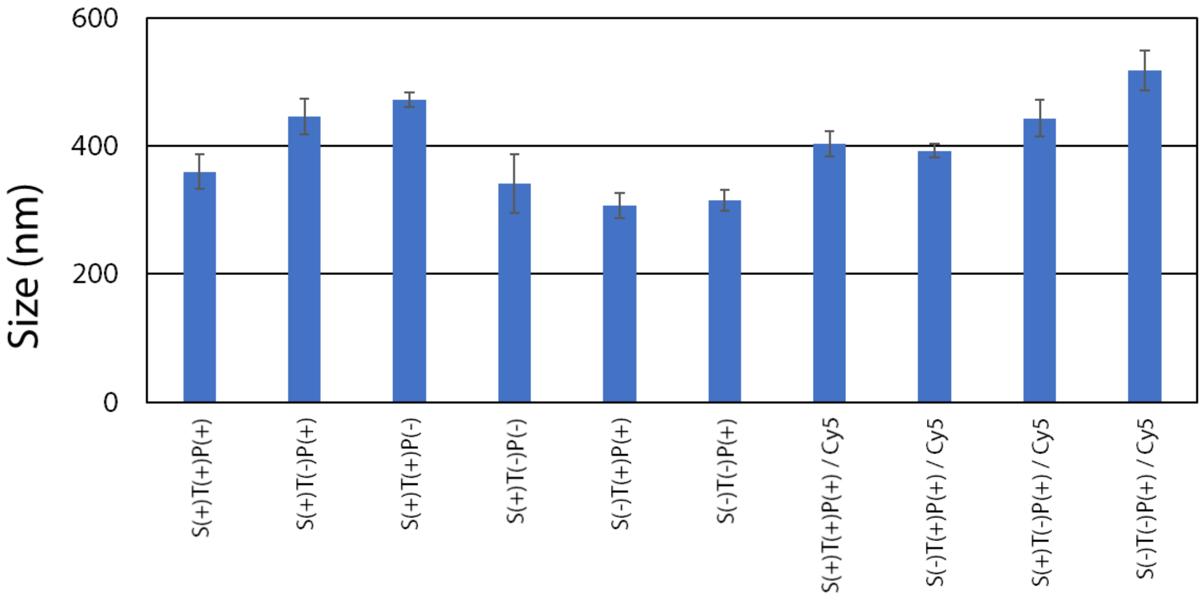

Supplementary Figure 5 DLS size measurement of various PRNCs

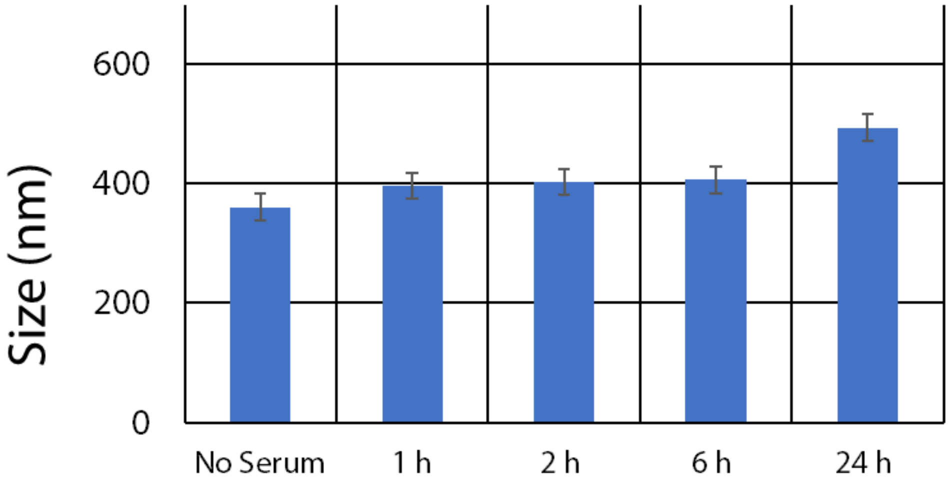

Supplementary Figure 6 Serum stability of PRNC

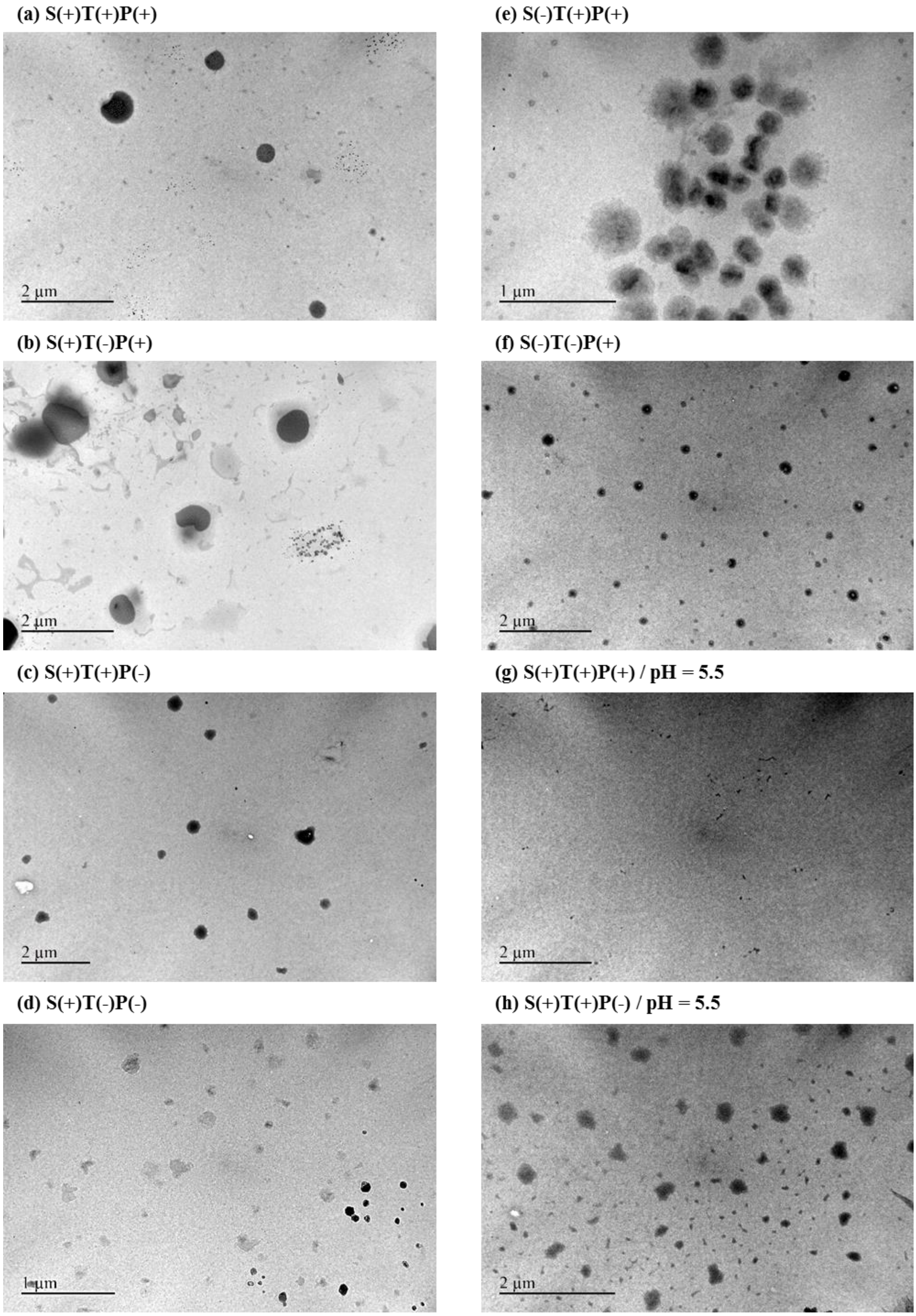

Supplementary Figure 7 TEM image of various PRNCs

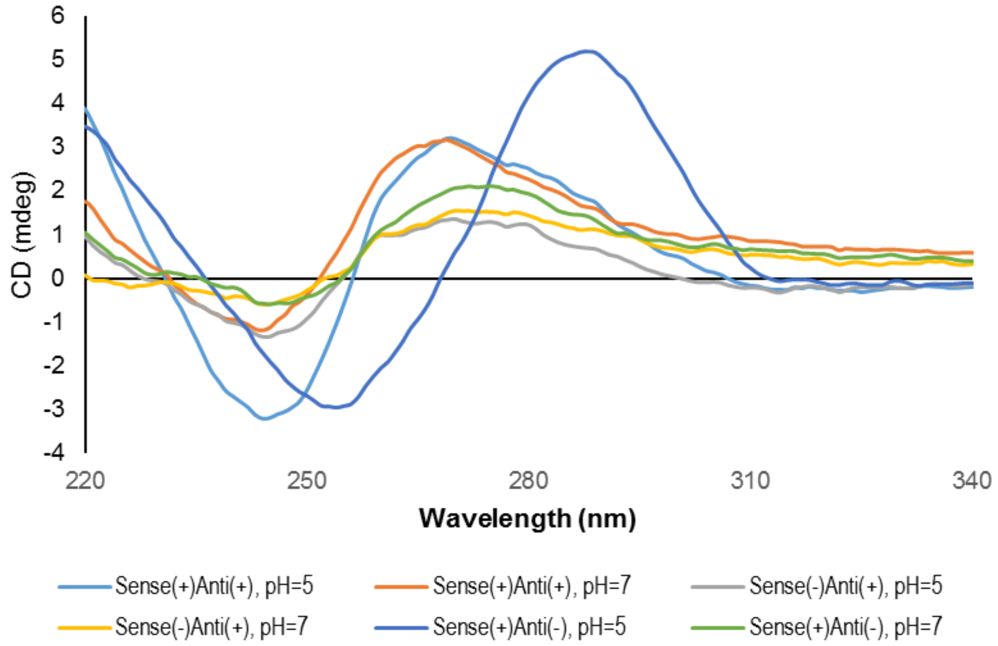

Supplementary Figure 8 CD spectra of i-motif DNA at different pH. Characteristic peak around 240 nm indicates the formation of i-motif structure in Sense(+) Anti(+) condition.

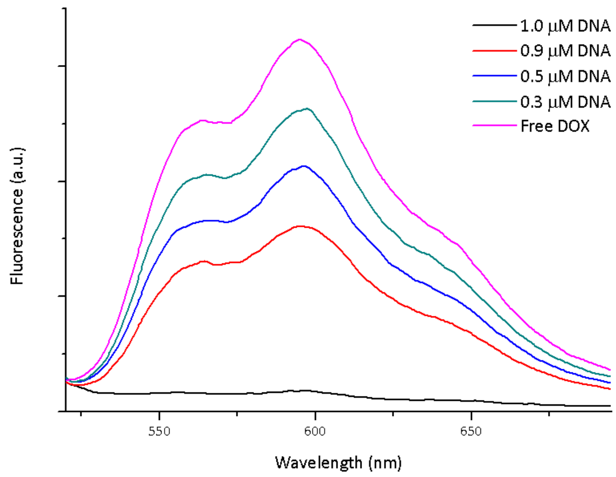

Supplementary Figure 9 Drug loading properties of PRNC. Fluorescent spectrum shows quenching of DOX fluorescence upon intercalation within dsDNA. Maximum 5 DOX molecule is loaded in single *i-motif* dsDNA.

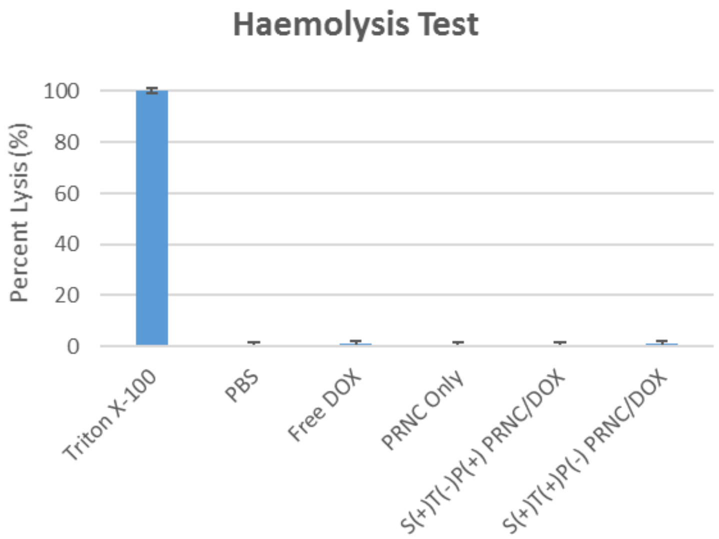

Supplementary Figure 10 Haemolysis test of PRNC and control groups. No significant lysis of red blood cell is observed in DOX loaded PRNCs.

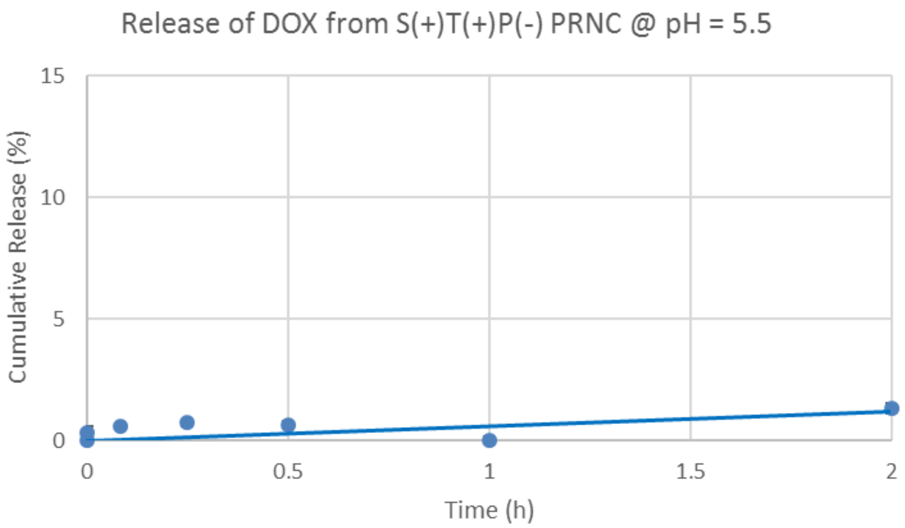

Supplementary Figure 11 Release of DOX from non-pH responsive control groups in acidic condition

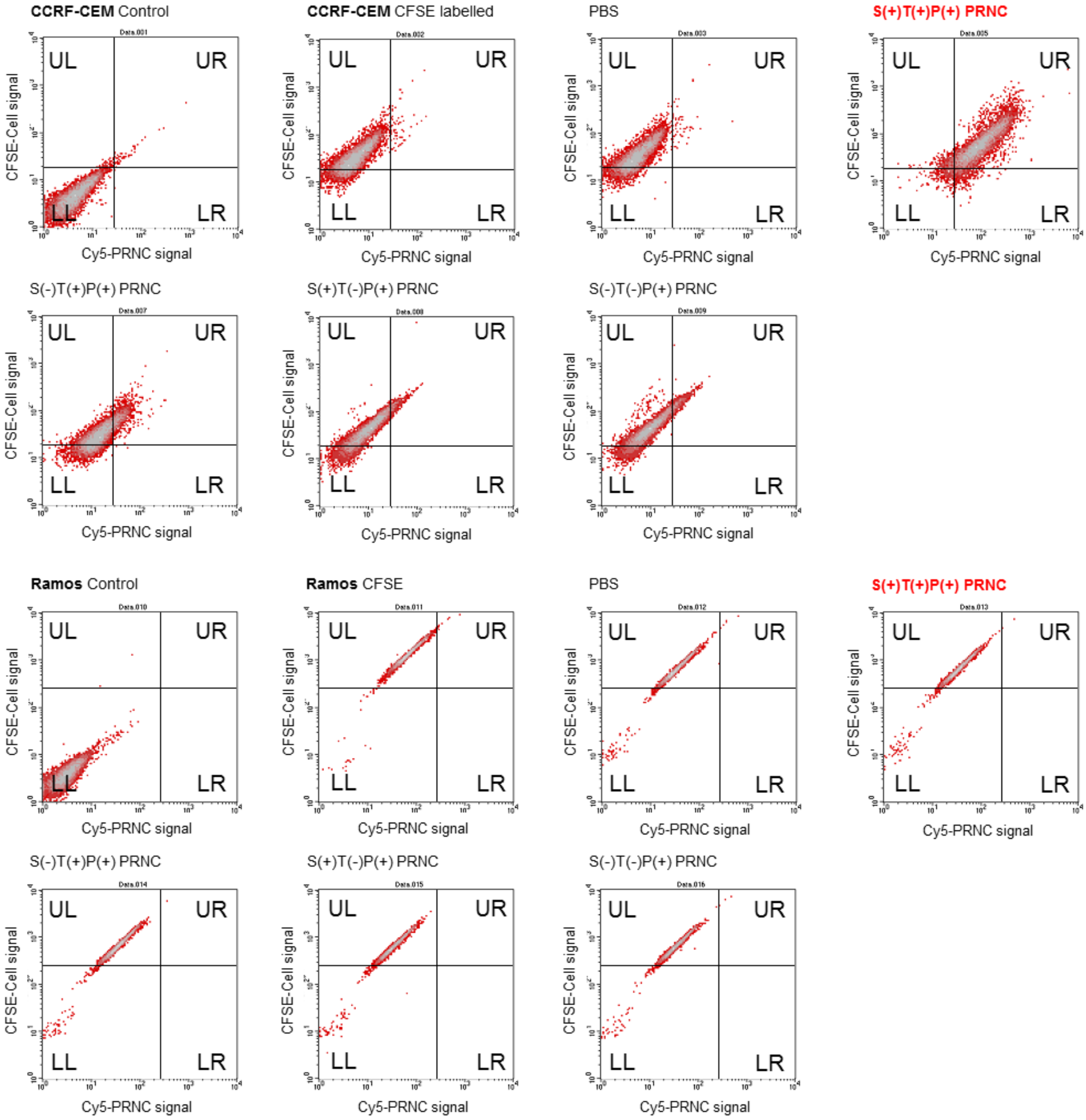

Supplementary Figure 12 Flow cytometry analysis results of CCRF-CEM transplanted mice treated with series of PRNCs

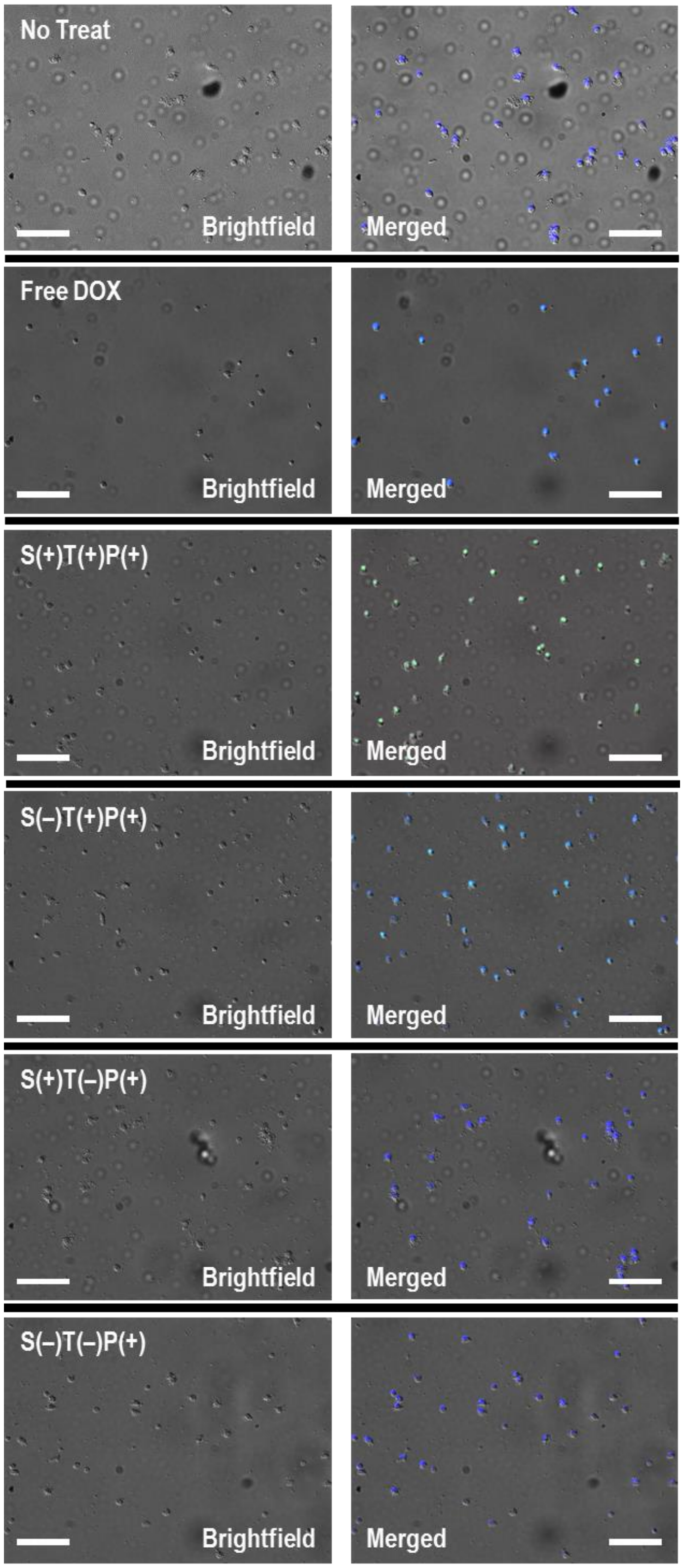

Supplementary Figure 13 Additional images of *in vivo* cytotoxicity evaluation. Brightfield and merged images of the microscopic samples identical to **Figure 5**. (Scale bar = 100 μm)

2. Supplementary Tables

Supplementary Table 1 Sequences of DNAs used in this experiment

Name	Sequence
DNA_i	5’ - AAA ACC CTA ACC CTA ACC CTA ACC C - 3'
$cDNA_i$	5’ - AAA AGC GTT AGC GTT AGG GTT AGG G - 3'
DNA_a	5’ - TTT TTA TCT AAC TGC TGC GCC GCC GGG AAA ATA CTG TAC GGT TAG A - 3'
sc_DNA_i	5’ - AAA ACG ATT CAC GGC TGT ACG ACA T - 3'
sc_cDNA_i	5’ - AAA AAT GTC GTA CAG CCG TGA ATC G - 3'
sc_DNA_a	5’ - TTT TAA ATG CGT CTT AGC AAT TAC GGA CAG TAG CAT CAC TTT AGT T - 3'

Supplementary Table 2 Types of PRNCs used in this experiment

Name	Details
S(+)T(+)P(+)	Sliding (+) / Targeting (+) / *i-motif* (+)
S(+)T(–)P(+)	Sliding (+) / Targeting (–) / *i-motif* (+)
S(–)T(+)P(+)	Sliding (–) / Targeting (+) / *i-motif* (+)
S(–)T(–)P(+)	Sliding (–) / Targeting (–) / *i-motif* (+)
S(+)T(+)P(–)	Sliding (+) / Targeting (+) / *i-motif* (–)
S(+)T(–)P(–)	Sliding (+) / Targeting (–) / *i-motif* (–)

Supplementary Table 3 IC50 values of various DOX loaded PRNCs towards CCRC-CEM and Ramos cells

Sample	IC50 value (µM DOX)	
	CCRF-CEM	Ramos
Free DOX	0.45	1.8
S(+)T(+)P(+)	0.4	2.4
S(+)T(–)P(+)	0.57	6.7
S(+)T(+)P(–)	0.75	N/A
S(+)T(–)P(–)	1.5	N/A
S(–)T(+)P(+)	1.25	N/A
S(–)T(–)P(+)	1.25	N/A

Supplementary Table 4 ROI fluorescence mean intensity calculation of microscopic images

Sample	DAPI	AnnexinV	PI	DAPI to AV	DAPI to PI
PBS	6352.89	496.9	802.98	8%	14%
DOX	11962.05	4276.95	340.96	36%	6%
S(+)T(+)P(+)	5607.61	6872.27	6938.39	123%	124%
S(-)T(+)P(+)	5911.65	2893.5	650.45	49%	12%
S(+)T(-)P(+)	10266.87	478.61	290.89	5%	5%
S(-)T(-)P(+)	3680.74	576.46	472.95	16%	8%
